# Multifunctional roles of γ-enolase in the central nervous system: more than a neuronal marker

**DOI:** 10.1186/s13578-024-01240-6

**Published:** 2024-05-12

**Authors:** Selena Horvat, Janko Kos, Anja Pišlar

**Affiliations:** 1 Department of Pharmaceutical Biology, Faculty of Pharmacy, University of Ljubljana, Aškerčeva 7, 1000 Ljubljana, Slovenia; 2 Department of Biotechnology, Jožef Stefan Institute, Jamova Cesta 39, 1000 Ljubljana, Slovenia

**Keywords:** γ-Enolase, Neuronal marker, Neurotrophic-like factor, Neurodegeneration, Neuroprotection

## Abstract

Enolase, a multifunctional protein with diverse isoforms, has generally been recognized for its primary roles in glycolysis and gluconeogenesis. The shift in isoform expression from α-enolase to neuron-specific γ-enolase extends beyond its enzymatic role. Enolase is essential for neuronal survival, differentiation, and the maturation of neurons and glial cells in the central nervous system. Neuron-specific γ-enolase is a critical biomarker for neurodegenerative pathologies and neurological conditions, not only indicating disease but also participating in nerve cell formation and neuroprotection and exhibiting neurotrophic-like properties. These properties are precisely regulated by cysteine peptidase cathepsin X and scaffold protein γ_1_-syntrophin. Our findings suggest that γ-enolase, specifically its C-terminal part, may offer neuroprotective benefits against neurotoxicity seen in Alzheimer's and Parkinson's disease. Furthermore, although the therapeutic potential of γ-enolase seems promising, the effectiveness of enolase inhibitors is under debate. This paper reviews the research on the roles of γ-enolase in the central nervous system, especially in pathophysiological events and the regulation of neurodegenerative diseases.

## Introduction

Enolase (EC 4.2.1.11) is one of the most ubiquitous and abundantly expressed proteins in the body and has a multitude of functions. It is prominently expressed in the cytosol in which its main function is serving as a glycolytic enzyme that catalyzes the conversion of 2-phosphoglycerate to phosphoenolpyruvate during glycolysis and the reverse reaction during gluconeogenesis [1]. This process is crucial as it facilitates the formation of high-energy compounds such as ATP and cofactor NADH, thereby providing the energy and materials necessary for cell metabolism [2]. Interestingly, enolase has also been found to have other moonlighting functions, which are not related to its primary function in glycolysis, making it a truly multifunctional protein. These additional roles are isoform-related and include the involvement of enolase in hypoxia tolerance [3, 4], tumor suppression [5, 6], and cell surface plasminogen binding [7, 8]. Moreover, when enolase is overexpressed in the lens, it also serves as a lens structural protein [9, 10]. Additionally, enolase acts as a DNA-binding protein [11] and a tubulin/microtubule-binding protein during myogenesis [12, 13]. The functions of enolase are not confined only to the cytosol. Enolase is also present in the nucleus, in which it is believed to participate in the regulation of genes governing cell growth and structural transformation [14, 15].

Enolase exists as a dimer comprising three distinct subunits: α, β, and γ. Each of these subunits performs specific regulatory functions and participates in various physiological and pathological processes, including those associated with the central nervous system (CNS) and neurodegenerative disorders. Specifically, α- and γ-enolase have been strongly associated with neurodegenerative conditions such as Alzheimer's disease (AD) and Parkinson's disease (PD) [16–18]. Particularly γ-enolase is a widely recognized and reliable biomarker for neuronal function. It shares many characteristics with neurotrophins, i.e., proteins that are essential for the development, survival, and function of neurons in the central and peripheral nervous systems [19]. In this review, we outline the functionality of the enolase enzyme family in the CNS, with an emphasis on the importance of γ-enolase as a biomarker for neuroinflammation-induced neurodegeneration. Furthermore, we examine the diverse functions of γ-enolase in the CNS, particularly its role as a neurotrophic-like factor in promoting neuronal growth, differentiation, and survival. Finally, we discuss the involvement of γ-enolase in neuroinflammation and neurodegeneration.

## The functional diversity of enolase

### Enolase isoforms in humans: from structure to function

**Fig. 1:**
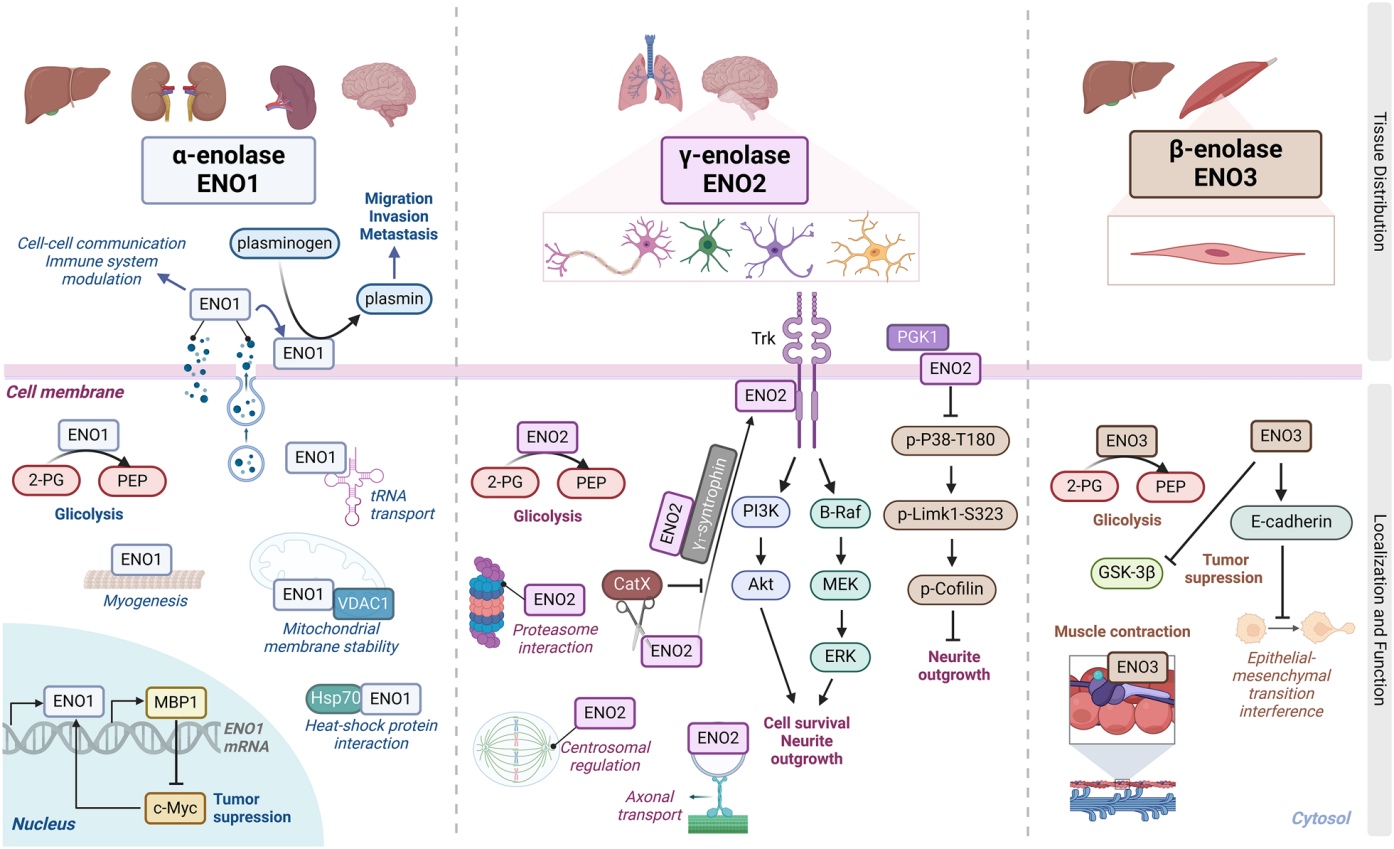
Tissue distribution, subcellular localization and functions of enolase isoforms. α-Enolase is present in most of tissues and found in the cytosol, cytoskeleton, nucleus, mitochondrial membrane, and cell membrane, performing roles in glycolysis, stress response, myogenesis, gene regulation, and acting as a plasminogen receptor. γ-Enolase is expressed in lung and nervous tissues and located in the cytosol and on the cell membrane, involved in glycolysis and neural development, but is absent from the nucleus. β-Enolase is found in liver and muscle tissues and present in the cytosol, engaging in glycolysis and interacting with sarcomeric troponin, affecting muscle contraction and the epithelial-mesenchymal transition (EMT) process [8, 13, 25, 31, 45, 58, 64, 66–83]

In humans, enolase exists in three distinct isoforms, also referred to as subunits. The first isoform, enolase 1, also known as α-enolase or non-neuronal enolase, is encoded by the *ENO1* gene and is involved in a variety of cellular processes [20]. The second isoform, enolase 2, also known as γ-enolase or neuron-specific enolase (NSE), is encoded by the *ENO2* gene and is predominantly expressed in neurons [1]. The third isoform, enolase 3, known as β-enolase or muscle-specific enolase, is encoded by the *ENO3* gene and is primarily found in muscle tissues [21]. In addition to these three isoforms, two other *ENO-*like genes have been reported in humans: *ENO4* (also called *ENOLL*) and *ENO5* (also called *ENOF1* or *ENOSF1*); however, they have been less studied [22]. All enolase isoforms primarily possess a glycolytic function [23, 24], and their expression is tissue-specific, where α-enolase, which is ubiquitously expressed in most tissues; β-enolase, which is primarily expressed in muscle tissue; and γ-enolase, which is mostly expressed in neural tissues (Fig. 1) [25]. Each isoform performs distinct functions depending on various factors such as developmental, metabolic, or pathophysiological conditions (Table 1).

**Table 1:** Overview of human enolase isoforms: tissue expression, functions, and clinical relevance

Isoform	Alternate name(s)	Enolase gene	Subunits	Tissue expression	Physiological functions	Clinical relevance	References
α-enolase	Enolase 1, C-myc promoter-binding protein, MBP-1, MPB-1, non-neural enolase, phosphopyruvate hydratase, plasminogen-binding protein	*ENO1* encodes α subunit	αα, αγ, αβ	Adipose, all neurological tissues, liver, spleen, and kidney	Role in intercellular communication, migration, and a pro-inflammatory phenotype of immune cells Protective effect by enhancing anaerobic metabolism as a hypoxic stress protein It interacts with microtubules during myogenesis, contractile filaments in cardiomyocytes during contraction, and the centrosome during the cell cycle. In its mitochondria-bound form, enolase acts as an RNA chaperone, binding and transporting nucleo-cytoplasmic tRNAs to mitochondria. It also contributes to stabilizing mitochondrial membrane potential by binding to VDAC1, an integral mitochondrial membrane protein	Prognostic and diagnostic cancer biomarker and oncotherapeutic target	[6, 13, 20, 22, 26–32]
γ-enolase	Enolase 2, neural enolase, neuron-specific enolase	*ENO2* encodes γ subunit	γγ, αγ	Neurons and neuroendocrine tissue, neuronal support cells	Promotes neuronal survival, differentiation, and axonal regeneration	Biomarker for small cell lung cancer and neuroendocrine tumors Confirmed upregulation in neurological conditions and neurodegenerative diseases	[1, 14, 22, 26, 33–42]
β-enolase	Enolase 3, muscle-specific enolase, skeletal muscle enolase	*ENO3* encodes β subunit	ββ, αβ	Muscle	Distinguishes proliferating myoblasts from various stages of development, indicating β-enolase as a marker of human myoblast heterogeneity Role in cholesterol metabolism due to accelerating hepatic cholesterol ester cumulation induced via the mediation of cholesteryl ester generation	Biomarker for myoblast heterogeneity that accompanies development Marker for identifying skeletal muscle injuries and the origin of bleeding Deficiency may lead to a rare inherited metabolic myopathy caused by an enzymatic defect of distal glycolysis Increased expression was detected in rhabdomyosarcoma tissue	[12, 21, 22, 26, 43–50]
Enolase 4	*ENOLL*	*ENO4*	/	Testis	Required for normal assembly of the fibrous sheath	/	[22, 23, 51]
Enolase 5	*ENOF1*	*ENO5*	/	Liver	May play a role in regulating the thymidylate synthase locus	/	[22, 24, 52]

There are five enolase isoforms: α-enolase (enolase 1), γ-enolase (enolase 2), β-enolase (enolase 3), enolase 4, and enolase 5, of which the last two have not been extensively investigated

The isoforms of enolase form functionally active dimers through non-covalent linkages, creating five different homodimers or heterodimers in which the monomers are oriented antiparallel to each other [53–55]. During development, the expression of these genes significantly changes. The ubiquitous isoform α-enolase (αα) can be replaced by muscle-specific β-enolase (ββ and αβ) or neuron-specific γ-enolase (γγ and αγ) [56].

All dimer combinations except βγ have been observed in vivo [57, 58]. The catalytic site of all human enolase isoforms consists of the conserved residues His158, Glu167, Glu210, Asp318, Lys343, Arg372, Ser373, and Lys396. Enolase requires the binding of the divalent metal ion Mg^2+^ for proper catalytic function [22, 59] and contains two ion-binding sites. Binding of the first Mg^2+^ ion induces conformational changes in the enzyme and enables substrate binding, whereas binding of the second Mg^2+^ ion is essential for forming a metal-ion-activated enzyme complex [54, 55]. The following residues are involved in the metal-binding sites: Ser40, Asp245, Glu293, and Asp318.

In addition, all enolase isoforms have a structure consisting of two main domains: the smaller N-terminal and the larger C-terminal domains [55, 60]. The N-terminal domain has a specific *β3α4* topology and contains a long flexible loop that folds back onto the active site [61]. It also includes a hydrophobic domain, which enables the enzyme to dock onto the surface of the plasma membrane [14, 62]. The C-terminal domain shows a *hββαα(βα)6* topology, which forms the active site of the enzyme. Between the two domains, each isoform possesses a characteristic short variable fragment that is situated predominantly on the surface of the molecule, away from the active site, and might be the region of contact with cytoskeletal or other cellular components [63]. The major functional difference between human isoforms is in their C-terminal parts. Whereas α- and β-enolase exhibit plasminogen-binding sites, formed by a lysine residue, the C-terminal part of γ-enolase has no lysine and does not bind plasminogen [64, 65]. The unique C-terminal structure of γ-enolase suggests a specialized function within the CNS, beyond its metabolic activity. Its abundant neuronal expression implies a role in maintaining the CNS functions. Consequently, this emphasizes the importance of continued research into γ-enolase, particularly as a promising candidate for therapeutic strategies in combating neurodegenerative conditions.

### Enolase isoforms cellular localization and related processes

Enolase isoforms exhibit functional diversity linked to their cellular localization, reflecting their roles in various cellular processes as summarized in Table 2. α-Enolase is ubiquitously present in the cytosol and is essential for glycolysis [25]. Moreover, it interacts with heat-shock proteins, including heat-shock protein 70 (Hsp70), suggesting a role in cellular stress responses [25, 66, 67]. It also associates with the cytoskeleton, contributing to myogenesis and stress-induced contraction [13, 31]. Moreover, its presence in the nucleus relates to the suppression of the c-myc gene, indicating a regulatory function in gene expression [68]. Additionally, α-enolase engages in RNA chaperone activity and is suggested to interact with voltage-dependent anion channel 1 (VDAC1). This interaction contributes to the stabilization of the mitochondrial membrane potential, which is essential for maintaining mitochondrial function [69, 70]. Furthermore, α-enolase is found on the cell surface and in the extracellular space, where it can either be associated with exosomes or secreted as a soluble protein [28]. When localized on the cell membrane, it acts as a plasminogen binding receptor, facilitating fibrinolysis, migration, invasion and metastasis, while localized in the extracellular space within exosomes, it participates in cell–cell communication and immune system modulation [8, 64, 71].

**Table 2:** Cellular distribution and related function of enolase isoforms

Isoform	Cellular distribution	Related cellular function	References
α-enolase	Cytosol	Glycolysis, heat-shock protein interaction	[25, 66, 67]
	Cytoskeleton	Myogenesis, stress-induced contraction	[13, 31]
	Nucleus	Suppression of c-myc	[68]
	Mitochondrial membrane	RNA chaperone activity, membrane potential stabilization	[69, 70]
	The outer side of the cell membrane	Plasminogen binding receptor	[8, 64, 71]
	Extracellular space (exosomes)	Plasminogen binding receptor	[8, 64, 71]
γ-enolase	Cytosol	Glycolysis, centrosome interaction; proteasome interaction	[58, 72, 73]
	Cytoskeleton	Integration and transport within the axonal cytoskeletal structure	[76]
	Nucleus	Absent	[74, 75]
	The inner side of the cell membrane	Cell survival, neurite outgrowth	[78–81]
	The outer side of the cell membrane	Neurite outgrowth	[77]
β-enolase	Cytosol	Glycolysis, sarcomere troponin interaction, interference with EMT process	[45, 82, 83]

Enolase isoforms exhibit diverse roles across specific subcellular localizations in mammalian cells highlighting the importance of understanding their related functions

γ-Enolase, predominantly located in the cytosol of brain cells, participates in glycolysis, but in a lesser extent than α-enolase [58]. Moreover, γ-enolase was reported to be interacting with centrosome and proteasome [72, 73], however is notably absent from the nucleus [74, 75]. Furthermore, γ-enolase was shown to be transported within axons as components of the slow component b complex, indicating a structured organization rather than free diffusion in the cytoplasm [76]. Recent findings also suggest its presence at the neural membrane, where it binds strongly with phosphoglycerate kinase 1 (Pgk1). This interaction significantly enhances the neurite outgrowth of motoneurons by reducing the signaling through p-P38-T180, p-Limk1-S323, and p-Cofilin pathways [77]. On the inner side of the cell membrane, γ-enolase supports cell survival and neurite outgrowth, highlighting its importance in neural development [78–81].

β-Enolase, localized in the cytosol of muscle cells, plays a key role in glycolysis and interacts with sarcomeric troponin, facilitating efficient energy metabolism and muscle contraction dynamics [82]. Moreover, β-enolase is also implicated in the epithelial-mesenchymal transition (EMT) process, where high expression of β-enolase is associated with increased levels of E-cadherin and the down-regulation of p-GSK-3β. This suggests that β-enolase may act as a suppressor by inhibiting EMT, indicating a potential role in cell differentiation and cancer progression [45].

Overall, the distinct subcellular locations of enolase isoforms reflect their multifunctional nature, revealing their involvement in a wide range of cellular processes (Fig. 1), from metabolism and stress response to cell structure, movement, and communication.

## NSE as a neuronal biomarker

### The diagnostic spectrum of NSE: from neurological disorders to neuroendocrine oncology

NSE is, due to its highly specific localization, recognized as a diagnostic and prognostic biomarker for tumors derived from neurons and peripheral neuroendocrine cells. It can provide quantitative measures of brain damage [84, 85], and NSE levels correlate with brain tumor size, the number of metastatic sites, and response to treatment. Additionally, NSE is currently recognized as the most reliable tumor marker for diagnosing, prognosing, and monitoring small-cell lung cancer [86]. NSE can be measured in cerebrospinal fluid (CSF) and serum [40, 42, 87]. In addition to CFS and serum NSE biomarkers, many studies have aimed to validate a plasma biomarker for neuronal damage that could be used non-invasively to study neurological diseases in both acute and chronic models [39, 88, 89].

Furthermore, after neuronal tissue damage, NSE levels rise sharply, correlating with injury severity and thus serving as a reliable biomarker for brain damage [42, 90]. The increase in NSE levels has been observed in some neurological states, including traumatic brain injury [91–94], ischemic stroke [95–100], seizures [101, 102], intracerebral hemorrhage [91, 92], cardiac arrest [103–107], spinal cord injury [108–112], AD, and PD [41, 113–116]. Research also indicates that after spinal cord injury (SCI), rat serum shows increased NSE levels compared to sham-operated animals [117]. Moreover, NSE levels in critically ill septic patients may be a promising biomarker for neuronal injury in sepsis [37, 118]. In addition, elevated NSE levels are also considered to be a potential prognostic biomarker for neuronal stress due to oxidative damage in several neurodegenerative pathologies [119, 120]. Oxidative stress prompts protein and nucleic acid oxidation, lipid peroxidation, and apoptosis, which lead to decreased brain function and loss of synapses and neurons [18]. Therefore, NSE is a robust, multi-faceted, and important biomarker for diagnosing and tracking the progression of numerous neurological conditions, monitoring tumor development, and evaluating cerebral tissue damage, thereby providing significant insights into complex neuronal processes. The release of NSE into the extracellular space, whether through secretion by healthy neurons or from those damaged in pathological states, is not established. However, the presence of NSE in the extracellular space, particularly at low concentrations (ranging from a few to 100 ng/mL), is proposed to have a neuroprotective impact, suggesting its increased levels during neurological disorders might contribute positively to the resilience and survival of affected neurons [19, 81].

### Limitations of using NSE as a neuronal biomarker

When using NSE as a neuronal biomarker, it is crucial to acknowledge its key limitations. First, the αγ subunit dimer of NSE can be present (in low amounts) in erythrocytes and platelets, suggesting that hemolysis could significantly influence the specificity of serum NSE [121]. A potential solution could be an introduction of a hemolysis correction factor or a preliminary hemolysis index [87, 122, 123]. Second, the prolonged half-life of NSE (in comparison to other neuronal biomarkers, such as S100β) can disturb short-term monitoring [40, 124]. Careful consideration should be given to the release patterns of NSE, as they can greatly impact optimal NSE sampling times. Finally, the availability of various NSE assays and varying cut-off points used for NSE measurements can make standardization across studies challenging [40, 41]. So far, the γ-enolase isoform of NSE has been recognized as a well-known neuronal marker with a less-known role in neuronal development and differentiation, which will be discussed further.

## The role of γ-enolase in brain cells

### The shift from α-enolase to γ-enolase expression in brain cells

In the mammalian brain, two distinct isoenzymes of enolase are present. The isoform α-enolase is ubiquitously expressed and is replaced by other isoforms during tissue development. Predominantly present during embryonic brain development, α-enolase levels decrease as neurons mature [125–127]. α-Enolase is a versatile protein, interacting with an array of cytoplasmic, nuclear, and membrane molecules, including various glycolytic enzymes (e.g., pyruvate kinase, phosphoglycerate mutase, and aldolase) [128]. Moreover, it binds to microtubule network proteins such as F-actin and tubulin [129]. The roles of α-enolase depend greatly on its intra- and extracellular localization. In the nucleus, it primarily regulates cell proliferation, differentiation, and metabolism [28]. Additionally, the *ENO1* gene, using a different transcription start codon, can produce a 37 kDa protein known as Myc Binding Protein-1, which functions as a tumor suppressor by inhibiting the activity of c-Myc [2, 15, 68]. The binding to plasminogen through α-enolase C-terminal lysine boosts plasminogen activation, triggering the activation of collagenases and degradation of extracellular matrix proteins and thus facilitating pathogenic invasions, inflammatory cell infiltration, and cancer cell migration and metastasis [8, 64].

The brain's second enolase isoform, γ-enolase, is 434 amino acids long and the most acidic enolase isoform. It has a subunit molecular mass of approximately 39 kDa, with the native form weighing 78 kDa, depending on the subunit combination [54]. Increasing γ-enolase expression is a gradual process that begins after neurogenesis in specific areas and slowly escalates to adult levels. A developmental study of the gene expression of enolase α- and γ- subunits in the rat brain showed that both subunits contribute to energy production in mature brain neurons, with enolase subunit compositions changing depending on the neuron type and maturation stage [130]. Studies on developing rat and rhesus monkey brains demonstrated the presence of α-enolase in neuron-producing zones, indicating a shift to γ-enolase during neuronal development [125]. Furthermore, a study of neurons in the cerebellum and neocortex has revealed that neurons are α-enolase-positive during migration and only become γ-enolase-positive once they have settled into their final location and have formed complete synaptic connections [126]. Thus, γ-enolase is intricately tied to the differentiation stage of cells and is a marker for mature neurons. Some migrating cells may contain hybrid enolase, and certain cell types may not fully convert to the γ-enolase type, even during the later stages of development [126].

### Localization and expression of γ-enolase in brain cells

γ-Enolase is primarily found in neurons and cells of the diffuse neuroendocrine system, specifically those belonging to the amine precursor uptake and decarboxylation lineage [131, 132]. It is involved in a variety of neuron-specific processes and plays a significant role in the physiology and pathology of the nervous system. In terms of neuronal differentiation and maturation, γ-enolase levels vary during the development of the brain. Its basal levels seem to be primarily regulated by mRNA levels during brain development [125, 127]. Besides being expressed specifically in fully developed neurons and cells originating from the nervous system, γ-enolase is also expressed in oligodendroglial cells during the transformation of precursors into mature oligodendrocytes. The enzymatic activity and protein and mRNA levels of γ-enolase in cultured rat oligodendrocytes are comparable to those found in cultured neurons. As differentiation progresses from oligodendrocyte precursors to mature oligodendrocytes, γ-enolase expression significantly increases, indicating its role in this transformation. Interestingly, in fully mature adult cells, γ-enolase expression is repressed, implying that its primary function is intricately tied to differentiation and maturation [133]. However, γ-enolase expression in oligodendrocytes is lower than that in neurons [133, 134]. According to previous studies using enzymatic and immunological techniques, the γ-subunit of enolase is present in cultured rat astrocytes, meningeal fibroblasts, and oligodendrocytes. Moreover, most enolase activity is attributed to the αα isoform in cultured neurons, astrocytes, fibroblasts, and oligodendrocytes. In culture, the sum of αγ and γγ enolase activities increases during maturation [58]. Moreover, γ-enolase demonstrates a significantly higher binding affinity to neurons than to astrocytes or fibroblasts, underscoring its unique physiological relevance to neurons [19].

### Glycolytic activity of γ-enolase in brain cells

Glucose is the primary and essential energy substrate for the adult brain. Glycolytic activity is the primary enzymatic function of γ-enolase, which is primarily located in cytoplasm and cytosol. The main role of γ-enolase is to exert glycolytic activity by converting glucose to pyruvate, corresponding to neuronal health and activity [135]. Despite its critical enzymatic function, initial studies indicated that the kinetic properties of γ-enolase, including its substrate affinity and reaction rates, are very similar to those of non-neuronal enolases [136]. However, γ-enolase distinguishes itself from other enolases by its marked structural stability under conditions that typically inactivate other enolase forms, such as high concentrations of chloride ions, elevated temperatures, and the presence of urea [137]. This stability is suggesting an evolutionary adaptation of γ-enolase to meet the specific metabolic demands of neurons and maintain glycolysis under physiologically stressful conditions.

The correlation between the glycolytic activity of γ-enolase and its neurotrophic effect appears to be minimal, as evidenced by the observation that low concentrations (ranging from 10^–9^ to 10^–8^ g/mL) of γ-enolase can elicit a neurotrophic response, whereas the enzymatic reaction substrate and product, namely 2-phosphoglycerate and phosphoenolpyruvate, do not influence neuronal viability. Additionally, the other two isoforms of enolase, αα and ββ, exhibit no impact on the viability of neurons, thereby underscoring the distinct function of γ-enolase in neuronal survival [81].

### The role of γ-enolase in brain development: beyond enzymatic activity

**Fig. 2:**
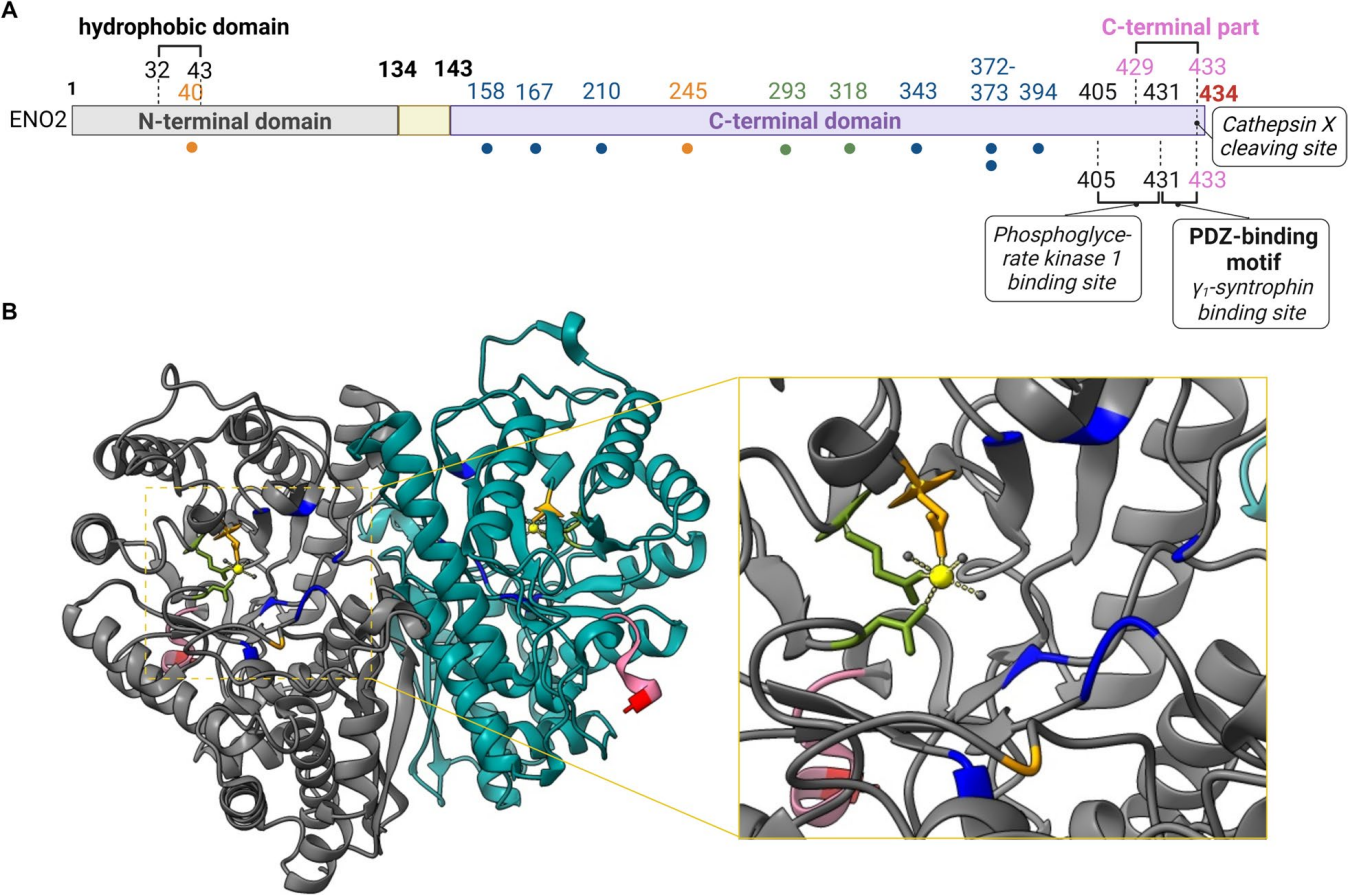
Domain structure and spatial conformation of γ-enolase. (**A**) The schematic representation of γ-enolase displays three distinct domains: an N-terminal domain (grey), a short variable domain (yellow), and a C-terminal domain (purple). It also displays the γ-enolase catalytic sites (blue), Mg^2+^-binding sites (orange), and sites serving both functions (green), which present predicted site for glycolic activity of the enzyme. The γ-enolase hydrophobic domain spans from 32A to 43Y, and the PDZ-binding motif spans from 431S to 433L (black brackets), latter representing γ_1_-syntrophin binding site. The γ-enolase C-terminal part (pink) consists of the last six amino acids, and the cathepsin X cleaving site (red) comprises the last amino acid at the C-terminus. Partly covering the C-terminal part, there is a proposed binding site of phosphoglycerate kinase 1. Features of γ-enolase (ENO2) were obtained from UniProt (P09104). The image was prepared with BioRender. (**B**) A ribbon model of the enzyme demonstrates the spatial conformation of γ-enolase, which is composed of two γ subunits (grey and turquoise). The catalytically active sites (blue, orange and green) with two Mg^2+^ ions (yellow) are proposed for glycolytic activity of γ-enolase. The C-terminal part of the molecule (pink) is believed to be important for neurotrophic activity and comprises the cathepsin X cleaving site at the C-terminus (red). The crystal structure of γ-enolase was obtained from Protein Data Bank (5TD9). The image was prepared by the authors with ChimeraX

The diverse cellular localization of γ-enolase, which depends on pathophysiological conditions, suggests that γ-enolase is involved in more than only glycolysis. The enzyme's active site, which is responsible for binding of the substrate 2-phosphoglycerate and consequently for glycolytic activity, is situated at the C-terminal domain (Fig. 2). Furthermore, γ-enolase can also be found in the plasma membrane, and extracellular space [75]. For example, γ-enolase is associated with the plasma membrane in neuronal and glial cells [62, 80, 138]. However, this association was observed under specific conditions, e.g., in neoplastic and non-neoplastic proliferating Schwann cells and serum-deprived neuron-like cells [75, 80]. Nevertheless, this association could be facilitated by the hydrophobic domain located in the N-terminal region (AAVPSGASTGIY at positions 32–43) [62]. Studies have shown that γ-enolase exhibits neurotrophic-like behavior when it is bound to the plasma membrane of neurons [80, 81].

The neurotrophic-like activity of γ-enolase is intrinsically linked to its specific localization within cells. In neurons, neurotrophic factors predominantly trigger two pathways essential for neuronal survival and axonal regeneration: the phosphoinositide 3-kinase (PI3K)/Akt and mitogen-activated protein kinase (MAPK)/extracellular signal-regulated kinase (ERK) pathways [139]. Specifically, the MAPK/ERK pathway, through activated Raf-dependent signaling, primarily drives axonal elongation, whereas the PI3K/Akt pathway, activated by active Akt, plays a crucial role in branching and increasing axonal caliber [140]. Takei et al. showed that in cultured neurons, highly purified γ-enolase supports survival in a dose-dependent manner [81]. The C-terminal part of γ-enolase, which comprises the last six amino acids, features a PDZ-binding motif (Fig. 2). This motif facilitates interactions with various PDZ-domain-containing proteins that participate in the intracellular redistribution of molecules and signaling pathways. In particular, scaffold protein γ1-syntrophin binds the C-terminal part of γ-enolase through its PDZ domain, and translocate it to the plasma membrane [80]. The proposed biologically active domain for the neurotrophic activity of γ-enolase is situated at the protein's C-terminal part [78]. Studies suggest that the C-terminal part of γ-enolase promotes neuronal survival, differentiation, and axonal regeneration by regulating signaling that depends on neuronal growth factor receptors [19, 78]. The receptor of the tropomyosin receptor kinase (Trk) family has been shown as a potential binding partner of γ-enolase and kinase activity of Trk is required for γ-enolase-mediated neurotrophic signaling (Fig. 1). However, the specific location of the neurotrophic site of γ-enolase (responsible for its neurotrophic activity) remains to be clearly defined. Recent studies have identified phosphoglycerate kinase 1 (Pgk1) as a novel binding partner for γ-enolase, suggesting that their interaction plays a crucial role in facilitating neurite outgrowth (Table 2) [77].

### Neurotrophic-like activity of γ-enolase peptide

Synthetic peptide mimicking the C-terminal part of γ-enolase promotes survival in rat neocortical neurons and exhibits neuroprotective action under low-oxygen conditions [19, 78]. Furthermore, this peptide, also known as γ-Eno peptide, exhibit neurotrophic properties, resembling those of neurotrophic factors that support cell survival and promote neurite outgrowth by activating the PI3K/Akt and MAPK/ERK pathways (Fig. 1). In agreement with this, γ-enolase activation of these pathways enhances SH-SY5Y cell survival and neurite growth [139]. Additionally, treatment of differentiated SH-SY5Y cells with γ-Eno peptide promoted Trk receptor internalisation and endosomal trafficking, as defined by reduced levels of Trk in clathrin-coated vesicles and increased levels in late endosomes. In this way, Rap1 is activated, which is required for neurotrophic activity mediated by γ-enolase. Additionally, the inhibition of Trk kinase activity by specific tyrosine kinase inhibitor revealed that increased SH-SY5Y cell survival and neurite outgrowth mediated by the γ-Eno peptide through activation of signaling cascade depends on Trk kinase activity [79]. Moreover, Hattori et al. showed distinct neurotrophic effects between γ- and α-enolase; specifically, γ-enolase demonstrated selective, dose-dependent, saturable, and calcium-dependent binding to neuronal surface manner, whereas specific binding of α-enolase to neurons was not detected [19]. In addition, γ-enolase possessed neuronal survival activity for the cultured neocortical neurons, whereas the effect was inhibited completely by antibodies targeting γ-enolase. Likewise, α-enolase isozyme had no effect on neuronal survival [81]. These findings provide insight into the neurotrophic-like action of γ-enolase and highlight its importance in promoting neurogenesis and providing neuroprotection.

### Regulation of the neurotrophic activity of γ-enolase

The activity of γ-enolase on neuronal survival and proper functioning relies on complex molecular interactions, among them a regulation mechanism has been shown for cathepsin X (a lysosomal cysteine peptidase) and γ_1_-syntrophin (a scaffold protein).

γ_1_-Syntrophin is a member of syntrophin family, known for its role as scaffold protein containing multiple protein‐protein and protein‐lipid interaction domains [141]. It is highly expressed in the brain where interacts with multiple proteins, including dystrophin (isoforms Dp71 and Dp140), diacylglycerol kinase-ζ and γ-enolase [80, 142, 143]. Among these interactions, through its PDZ domain, γ_1_-syntrophin interacts with γ-enolase, facilitating the translocation of γ-enolase to the plasma membrane, where γ-enolase exerts its neurotrophic activity. Silencing the γ_1_-syntrophin gene can significantly reduce the re-distribution of γ-enolase to the plasma membrane and impair its neurotrophic effects. Extensive co-localization of γ_1_-syntrophin and γ-enolase in neurite growth cones was shown in differentiated SH-SY5Y cells, indicating the importance of γ_1_-syntrophin in facilitating the neurotrophic activity of γ-enolase by ensuring its localization to the plasma membrane [80].

Cathepsin X, alternatively referred to as cathepsin Z, is a carboxypeptidase. Its exopeptidase activity impacts several molecular targets, including the β-chain of integrin receptors, γ-enolase, chemokine CXCL-12, bradykinin and kallidin, huntingtin and profilin 1 [144]. Cathepsin X has been shown to cleave two amino acids at the C-terminal part of γ-enolase, thus preventing it from binding with the γ_1_-syntrophin PDZ domain and disrupting its translocation to the plasma membrane [138, 145]. Hence, γ-enolase and its regulatory mechanisms are garnering growing scientific interest. The role of cathepsin X in neuritogenesis is largely linked to its proteolytic activity, with γ-enolase being one of its targets. The proteolytic activity of cathepsin X results in C-terminal cleavage of γ-enolase, affecting its neurotrophic activity. The interplay between cathepsin X and γ-enolase was demonstrated by a correlation between the high proteolytic activity of cathepsin X and the C-terminal cleavage of γ-enolase [146].

Moreover, potent selective irreversible (AMS36) and reversible (Z7) inhibitors of cathepsin X decreased the viability of patient-derived glioblastoma multiforme cells in vitro as well as macrophages and microglia cultured in their conditioned media. Moreover, research has indicated that cathepsin X and γ-enolase are co-localized in glioblastoma multiforme tissues, especially in macrophages and microglia [146]. These findings suggest that cathepsin X plays a role in the progression of glioblastoma multiforme and is a potential target for therapeutic interventions against this aggressive brain tumor. Furthermore, co-localization of cathepsin X and γ-enolase was demonstrated in a transgenic mouse model of AD, specifically, in aged Tg2576 mice that develop amyloid-β (Aβ) plaques in the brain. This was further validated in vitro in microglial cell cultures treated with Aβ peptide [147]. These accumulated findings underscore the significance of the interplay between γ-enolase and cathepsin X.

**Fig. 3:**
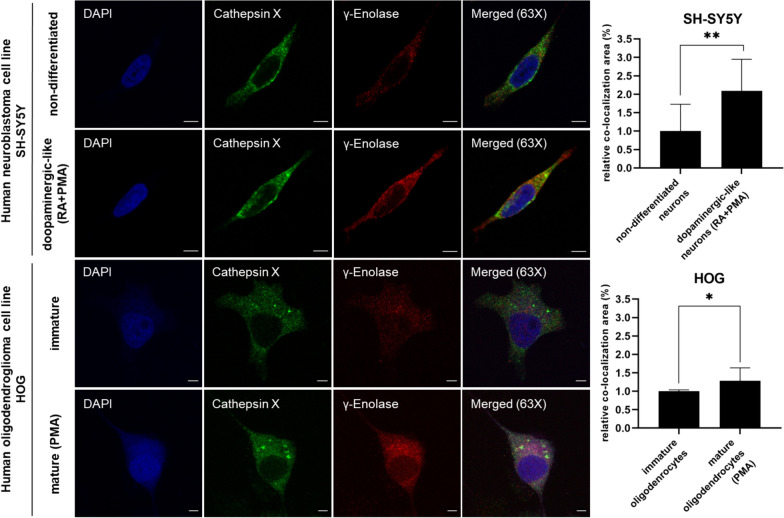
Co-localization of cathepsin X and γ-enolase in differentiated neurons and mature oligodendrocytes. Representative images (left) and quantification (right) of double immunofluorescence staining for cathepsin X (green) and γ-enolase (C-terminal part; red). Nuclei were counterstained with DAPI (blue). Human SH-SY5Y neuroblastoma cells were differentiated with retinoic acid (RA; 10 μM) in combination with phorbol myristate acetate (PMA; 80 μM) for 7 days in reduced serum media (RSM; 2% fetal bovine serum and 0.25% penicillin/streptomycin in DMEM/F12), whereas non-differentiated cells were treated with dimethyl sulfoxide (DMSO; 10 μM) as control. Human oligodendroglioma cell line (HOG) was differentiated to the mature state with PMA (100 nM) for 4 days in RSM, whereas non-mature cells were cultured in RSM. We observed higher γ-enolase expression and co-localization with cathepsin X in either differentiated or mature states. Images were acquired using an LSM 710 Carl Zeiss confocal microscope and ZEN imaging software. Data are presented as means ± SD of the pixels in the third quadrant of the scatter plot (cell numbers ≥ 10, n = 2) (two-tailed t-test, *P < 0.05, **P < 0.01). Scale bars: 5 μm

However, the exact role of γ-enolase in the maturation and differentiation of neurons and glia cells, along with its regulation by cathepsin X, remains to be determined. Based on our preliminary findings, the co-localization of these proteins appears to be greater in dopaminergic-like differentiated neurons compared to non-differentiated cells. Moreover, we observed increased co-localization in mature oligodendrocytes compared to immature cells (Fig. 3). Ongoing research is investigating the specific role of cathepsin X in neuritogenesis and possible implications for treating various neurological conditions. Our initial observations, together with our prior studies, where we showed colocalization of γ-enolase with cathepsin X [138, 147, 148], suggest an interaction between γ-enolase and cathepsin X. This insight could provide the basis for future research to investigate their potential roles in the mechanisms of neurological diseases.

## Significance of γ-enolase in neurodegeneration and neuroinflammation

### Neurodegeneration and neuroinflammation: insights from cathepsin X

Inflammatory responses are commonly observed and play a significant role in the advancement of ageing and neurodegenerative diseases (e.g., AD and PD) [149]. In a healthy state, homeostasis of the CNS is maintained by microglia and astrocytes. However, during neuroinflammation, these cells become chronically activated, leading to altered functioning and progression of neurodegenerative disorders [17]. Cathepsin X has been identified as a significant inflammatory mediator in neuroinflammation. Its expression and activity escalate rapidly under conditions of neuroinflammation-induced neurodegeneration [150]. Its overexpression was detected in various brain areas, such as the cerebral cortex, corpus callosum, subventricular zone, and external globus pallidu*s*. Importantly, this overexpression was mainly found in activated microglia and reactive astrocytes [150]. Moreover, continuous administration of a selective cathepsin X inhibitor, AMS36, demonstrated protective effects against neuroinflammation-induced striatal degeneration in a rat model. This was evidenced by the attenuation of lipopolysaccharide-mediated dilation of the lateral ventricles and a partial decrease in the extent of striatal lesion. These findings indicate that cathepsin X is a pathogenic factor and thus a potential therapeutic target in neuroinflammation-induced neurodegeneration [150]. Therefore, to ensure proper neuronal function, it is particularly important to precisely regulate the activity of cathepsin X [151].

Moreover, inhibiting cathepsin X may enhance the neurotrophic activity of γ-enolase. Suppressing cathepsin X in BV2 cells (a model of activated microglia) increased the levels of the intact form of γ-enolase [148]. Furthermore, pre-treating microglial cells with a specific cathepsin X inhibitor not only counteracted the decreased levels of active γ-enolase in the supernatant of lipopolysaccharide-activated microglia but actually increased the levels of the active γ-enolase form, which possesses neurotrophic and neuroprotective properties [148]. Therefore, cathepsin X represents a promising therapeutic candidate for tackling neuroinflammatory ailments.

### Protective potential of γ-enolase against neurotoxicity in AD and PD

Considering that γ-enolase is a target of cathepsin X, their relationship could be significant in neurodegenerative diseases linked with neuroinflammation. This is potentially applicable in in vivo animal models and in vitro cellular models of neurodegenerative diseases. The model of AD involves exposure to neurotoxins, particularly Aβ oligomers and fibrils that induce cell death, thereby mimicking in vivo neurodegeneration [152]. Aβ accumulation in the brain can lead to neurotoxicity, contributing to the progressive neuronal damage seen in AD [153]. Moreover, Aβ-induced toxicity is abolished in the presence of the active C-terminal peptide of γ-enolase, as evidenced by the levels of cell viability, lactate dehydrogenase release, sub-G1 cell population, and intracellular reactive oxygen species [154]. This suggests that γ-enolase, or more specifically its active C-terminal peptide, may have the potential to protect neurons against the neurotoxic effects of Aβ, such as those seen in AD. However, further research is needed to fully understand the mechanism by which γ-enolase exerts this protective effect and to determine whether this finding can be translated into effective therapeutic strategies for AD and other conditions characterized by Aβ-induced neurotoxicity. Furthermore, γ-enolase is upregulated in microglial cells surrounding Aβ plaques in Tg2576 transgenic mice overexpressing Aβ precursor protein (a model of AD) and plays a neuroprotective role in Aβ-related neurodegeneration that is regulated by cathepsin X. These findings suggest that γ-enolase participates in the regulation of neuronal survival and death in the context of AD [147].

In both in vivo and in vitro models, the characteristic nigrostriatal degeneration of PD is often simulated using the widely used neurotoxin 6-hydroxydopamine, a hydroxylated analogue of the endogenous neurotransmitter dopamine [155]. Previous studies have suggested that γ-enolase plays a role in maintaining and repairing damaged neurons. For instance, in a rat model of levodopa-induced dyskinesia (a motor complication that arises in PD patients after prolonged treatment with levodopa), γ-enolase levels were increased in dyskinetic animals compared to non-dyskinetic and bromocriptine-treated animals. This observation led to the proposal that upregulated γ-enolase may indicate the activation of cellular defense mechanisms in the dyskinetic striatum [156]. Another in vivo study examined the localization and activity of cathepsin X in a 6-hydroxydopamine-induced PD rat model. After 6-hydroxydopamine injection, cathepsin X expression and activity rapidly increased in the ipsilateral substantia nigra pars compacta, peaking after 12 h, and remaining strongly upregulated for at least 4 weeks post-injection. Initially, increased cathepsin X levels were localized in lysosomes in dopaminergic neurons that were primarily positive for tyrosine hydroxylase. After 12 h, only a few activated microglial cells showed positivity for cathepsin X. After 4 weeks, upon complete loss of dopaminergic neurons, cathepsin X upregulation persisted in activated glial cells. These findings suggest that cathepsin X upregulation could act as a pathogenic factor in PD. Therefore, inhibiting cathepsin X expression or activity might protect the nigrostriatal dopaminergic projection in PD, representing a new potential therapeutic target [157].

The roles of γ-enolase and its possible regulation by cathepsin X in neuroinflammation and neurodegeneration, alongside their interaction, underscore the potential for developing innovative therapies for neurodegenerative diseases such as AD in PD.

### Diverse Roles of γ-Enolase in neuroinflammation

Research in the field of neuroinflammation reveals that γ-enolase exhibits diverse roles, actively participating in both promoting and reducing neuroinflammatory responses. Furthermore, there is evidence to suggest that γ-enolase might both promote neuroinflammation and facilitate neuroprotection in spinal cord injury (SCI) among other neurodegenerative conditions [26, 158]. These studies indicate that increased γ-enolase expression and activity could escalate inflammation in the spinal cord after SCI, leading to more damage after the initial SCI [26]. To determine the role of γ-enolase, researchers employed the unique small-molecule inhibitor of enolase, ENOblock. This compound is reported to bind directly to enolase, inhibiting its activity [159]. Evidence suggests that inhibiting enolase might be a promising therapeutic approach for SCI, as it regulates metabolic hormones and decreases γ-enolase serum levels, inflammatory cytokines and chemokines, MMP-9 activation, and glial activation after SCI [117, 160]. Nonetheless, the exact role of γ-enolase after SCI and its potential effects on neurodegeneration remain unclear [26]. Conversely, another study noted that ENOblock did not inhibit α- or γ-enolase in in vitro assays and lacked selective toxicity against ENO1-deleted cancer cells [161]. These findings warrant further exploration of the effect of ENOblock on γ-enolase. Although certain research demonstrates that increased γ-enolase expression post-SCI intensifies inflammation and subsequent damage [117], the broader implications of its activity, particularly concerning central disorders (AD or PD) versus peripheral disorders (SCI), remain undetermined. Thus, more extensive research is crucial to discover the multifaceted role of γ-enolase across diverse neurological conditions, which could offer insights into its influence on disease progression and therapeutic value.

Furthermore, a significant amount of research has focused on the role of cathepsin X in neurodegeneration. This enzyme, along with other cysteine cathepsins, promotes neuroinflammation-driven neurodegeneration, especially due to its heightened activity in activated microglia [162]. Although cystatins have potential therapeutic effects, their effects are too broad, and thus researchers are exploring synthetic selective inhibitors to potentially renovate treatments for neurodegenerative conditions [163]. Our research indicates that AMS36, a cathepsin X inhibitor, effectively reduces the release of nitric oxide, a marker of activated microglia, without altering basal levels [148]. Moreover, AMS36 decreases lipopolysaccharide-induced IL-6 and TNF-α levels, underscoring the therapeutic potential of targeting cathepsin X in neuroinflammation-driven neurodegeneration. Additionally, in the presence of 6-hydroxydopamine, the effect of cathepsin X on the NF-κB pathway is evident; AMS36 halts both neurotoxin-induced NF-κB nuclear translocation and the degradation of its inhibitor IkBα [164]. Furthermore, cathepsin X has a notable regulatory effect on the MAPK signaling pathway in microglia through its proteolytic activity. AMS36 also effectively suppresses lipopolysaccharide-induced BV2 cell activation, significantly decreasing cytokine release from BV2 cells [148]. Overall, although cathepsin X has been identified as a potential target in neurodegeneration, the mechanism of its degenerative action, specifically the regulation of its target γ-enolase in brain cells, remains undetermined.

## Concluding remarks

To conclude, the glycolytic enzyme γ-enolase, beyond its acknowledged use as a biomarker, is gaining recognition as an important factor in physiological and pathological processes of the nervous system. It is implicated in a multitude of neuron-specific processes, including neuronal differentiation, maturation, and survival, as evidenced by its expression during brain development. Moreover, γ-enolase has been recognized as a neurotrophic-like factor, exhibiting a dynamic response to injury, disease, and changes in the microenvironment. However, the diverse functions of γ-enolase in the CNS, particularly its role in neuroinflammation and neurodegeneration, warrant further investigation. Understanding these processes together with tools that can regulate γ-enolase in neurons and glia might provide new opportunities for the treatment of CNS injury. The divergent opinions on the role of γ-enolase emphasize the importance of better understanding whether it acts as a pro-inflammatory, anti-inflammatory, or perchance both. This leads to the intriguing possibility that γ-enolase could simultaneously contribute to both neuroinflammation and neuroprotection in different neurodegenerative conditions. Therefore, it would be interesting to assess the effects of γ-enolase in neurons and activated glia cells in future studies. Furthermore, it is still unclear whether the molecular pathways involved in normal neural development and axon degeneration in neurodegenerative disease are the same or distinct. Potential therapeutic strategies involve addressing the effect of cathepsin X on γ-enolase. Specifically, the action of cathepsin X on the C-terminal dipeptide of γ-enolase inhibits its functions linked to neuronal survival and neuritogenesis. Utilizing cathepsin X inhibitors or peptides derived from the C-terminal of γ-enolase may amplify the neurotrophic benefits mediated by γ-enolase. In summary, γ-enolase extends beyond its well-known metabolic function; it plays a crucial role in neuronal development and neuroprotective processes, positioning it as a promising candidate for therapeutic intervention in neurodegenerative disorders. Further research is necessary to fully understand its functions and therapeutic possibilities within the CNS.

## Data Availability

Not applicable.

## Abbreviations

AD: Alzheimer's disease
Aβ: Amyloid-β
CNS: Central nervous system
CSF: Cerebrospinal fluid
EMT: Epithelial-mesenchymal transition
ERK: Extracellular signal-regulated kinase
HOG: Human oligodendroglioma cell line
Hsp70: Heat-shock protein 70
MAPK: Mitogen-activated protein kinase
NSE: Neuron-specific enolase
PD: Parkinson's disease
Pgk1: Phosphoglycerate kinase 1
PI3K: Phosphoinositide 3-kinase
PMA: Phorbol myristate acetate
RA: Retinoic acid
RSM: Reduced serum media
SCI: Spinal cord injury
Trk: Tropomyosin receptor kinase
VDAC1: Voltage-dependent anion channel 1
